# *Announcement*: National Campaign to Prevent Falls in Construction — United States, 2017

**DOI:** 10.15585/mmwr.mm6616a5

**Published:** 2017-04-28

Falls are a leading but preventable cause of death in the construction sector, accounting for 350 (37%) of the 937 fatalities among construction workers recorded in 2015 (*1*). Falls to a lower level from roofs, ladders, and scaffolds comprise 72% of construction fall deaths (*2*). During 2011–2015, roofing contractors reported the largest number of fall-related deaths (332) in this high-risk industry (*2*). Robust employment in the construction industry during 2000–2008 was followed by a considerable decline during the economic downturn of 2008–2012 (*3*). Since 2012, when employment levels were at their lowest (*4*), there has been an approximate 10% increase in employment in the construction industry. A total of 9.9 million U.S. workers were employed in construction during 2015. Small businesses with fewer than 20 employees account for 92.5% of all construction establishments, and 41.4% of all construction employees work in small businesses (*3*).

CDC’s National Institute for Occupational Safety and Health (NIOSH) works with construction sector partners to improve workplace safety through a government-labor management partnership. One product of this partnership is an annual national fall prevention campaign aimed at construction contractors, onsite supervisors, and workers. During May 8–12, the federal Occupational Safety and Health Administration and partners, including NIOSH, will host the National Safety Stand-Down to Prevent Falls in Construction. The voluntary “stand-down”* is an activity within the campaign, and provides an opportunity for construction employers to speak directly to their employees, including their non-English–speaking employees, about fall hazards and reinforce the importance of fall prevention. Participation in both the stand-down and falls campaign is strongly encouraged throughout the United States, including by state agencies, public health practitioners, and private contractors. Additional information is available at https://www.osha.gov/StopFallsStandDown/.

## Footnotes

* The term "stand-down" is taken from the U.S. military: whenever a death or a number of similar nonfatal injuries occurs, the military stops (i.e., stands down) all relevant activities while safety training is provided across all units engaged in the activity.
